# Point of Care Assessment of Sexual Concerns among AYA Oncology Active Patients and Survivors

**DOI:** 10.21203/rs.3.rs-2986799/v1

**Published:** 2023-05-31

**Authors:** Molin Shi, Karen J. Meltzer, Alexandra Dunker, Brittany C. Hall

**Affiliations:** The University of Texas Southwestern Medical Center, Department of Psychiatry, Division of Psychology; The University of Texas Southwestern Medical Center, Department of Psychiatry, Division of Psychology; The University of Texas Southwestern Medical Center, Moncrief Cancer Institute; The University of Texas Southwestern Medical Center, Department of Psychiatry, Division of Psychology

**Keywords:** AYA, adolescent and young adult oncology, sexual health, needs assessment, unmet needs

## Abstract

**Purpose:**

Adolescent and young adults (AYAs) oncology populations have unique sexual health concerns that deserve more attention. The current study aimed to describe the prevalence and characteristics of sexual health and related concerns in AYAs in active treatment and survivorship to move toward integrating sexual health in routine care.

**Methods:**

A total of 127 AYAs (ages 19–39) in active treatment and survivorship were recruited from three outpatient oncology clinics. In addition to providing demographic and clinical information, they completed an adapted version of the NCCN Distress Thermometer and Problem List (AYA-POST; AYA-SPOST) as part of an ongoing needs assessment study.

**Results:**

Over one quarter (27.6%) of the total sample (*M*_*age*_ = 31.96, SD = 5.33) – 31.9% of active treatment, and 21.8% in survivorship – reported at least one sexual health concern (i.e., sexual concern, loss of libido, pain with sex, and unprotected sex). The most frequently endorsed concerns differed between active treatments and survivorship. Both genders often endorsed general sexual concerns and loss of libido.

**Conclusion:**

The literature on sexual concerns in the AYA population is sparse and inconclusive, especially accounting for gender and other types of concerns. The current study highlights the need for further examination between treatment status, psychosexual concerns, emotional distress, and demographic and clinical factors. Given the prevalence of sexual concerns in AYAs in active treatment and survivorship, providers should consider integrating assessment and discussion of these needs at onset of diagnosis and as part of monitoring.

## Introduction

### Cancer and Sexual Health

The sexual health and functioning of patients across the cancer continuum is gaining more attention in both research and clinical practice [1], [2]. Sexual health can be directly and indirectly affected by the disease itself, treatments, and side effects from the interplay between physical, psychological, and interpersonal changes [3]. Sexual dysfunction are common distressing sequelae across cancer types [3], [4] and can include reduced libido, erection issues, difficulties with orgasm or pleasure, vaginal dryness, and painful coitus [3], [5]. Additionally, the following cancer-related experiences can negatively impact sexual well-being: traumatic stress, anxiety, depression, body image concerns, communication difficulties with partners, and guilt about changes in sexual functioning [5], [6]. Sexual well-being is an important aspect of quality of life [7]. In patients with cancer, higher sexual satisfaction is associated with lower psychological and physical symptom burden [8].

### AYA and Sexual Functioning

Cancer incidence rates among adolescents and young adults (AYAs, ages 15–39) is on the rise [9]. A cancer diagnosis for AYAs can lead to physical (e.g., pain, scarring, erectile dysfunction) and emotional (e.g., body dissatisfaction) changes that can disrupt the typical development of intimacy and sexuality at this stage [10] and a sense of missing out on these key milestones [11]. More than half of AYA active-treatment patients in a longitudinal study found sexual functioning to be problematic across a two-year period, with increased probability of endorsing sexual dysfunction over time [12]. Between 42–52% of AYAs with cancer report at least one sexual problem [12], [13]. Almost half of AYAs report problems with sexual functioning one year post-diagnosis and of those, 70% perceive continued negative effects from cancer treatment on sexual functioning at two years post-diagnosis [14]. Qualitative interviews with AYA cancer survivors revealed many perceive themselves as “damaged goods” and were hesitant toward sex and disclosure of their cancer history to potential sexual/romantic partners [15] and question their ability to please (potential) partners [11].

Despite the known sexual concerns among AYAs with cancer, this population often cites sexual issues among their significant unmet needs (e.g., [16], [17]). Specifically, AYA survivors have reported not feeling adequately informed by their oncology providers about cancer and treatment impact on sex drive, sexual side effects, and sexual well-being [11], [18]. The complex psychosexual factors are often understudied or avoided in clinical practice [19], the latter of which comes partly from oncology clinicians’ general lack of knowledge/experience in broaching these topics, personal discomfort and perceived discomfort from AYA patients, insufficient time, low priority of concern [20]. Patients can perceive their providers are more focused on surviving cancer than quality of life [11].

### Assessment of Sexual Concerns in AYAs

AYAs may have little knowledge about sexual health prior to their cancer diagnosis. Thus, they may experience confusion or embarrassment about cancer-related changes in functioning and feel uncomfortable broaching the topic with their healthcare provider [21]. Researchers and clinicians consistently emphasize the importance of oncology providers initiating the conversation about sexual health with this population [16] and oncology providers working with AYAs have emphasized the importance of using patient self-report measures to improve sexual health communication[22]. However, sexual health concerns continue to go unassessed and unaddressed by oncology providers [2]. One key barrier is the relatively scant relevant assessment tools for sexual health in cancer patients [23]. While some gold standard sexual health measures exist and have been validated with oncology populations (e.g., The Female Sexual Function Index, International Index of Erectile Dysfunction, Medical Outcomes Survey Sexual Functioning Scale, and PROMIS Sexual Functioning and Satisfaction Measures), they were not developed specifically for AYAs [1], [24]–[26]. Data on AYA sexual health is typically gathered as part of a generic psychosocial needs assessment survey [16] but few commonly-used psychosocial measures have been validated specifically for use in the AYA population [27]. Lastly, existing data on sexual concerns in this population was collected through mailed or electronic surveys, rather than as part of a healthcare visit (see Wettergren et al., 2017 [14] and most studies in Lehmann et al., 2022 and Cherven et al., 2021 [16], [28]). This reflects a gap in the literature regarding the assessment of AYA sexual health needs at the time of an oncology visit with an AYA-specific screening tool.

### Present Study

As noted above, AYAs experience a substantial burden of sexual dysfunction and a lack of adequate provider assessment of sexual functioning using AYA-specific tools. Thus, there is a growing attention to considering sexual health concerns as part of the well-being of AYA oncology populations, therefore movement toward integrating assessment and intervention of AYA sexual health in the context of routine medical care.

This exploratory study utilized a cancer needs assessment screening tool uniquely designed for AYAs in both active treatment and survivorship that asked about sexual concerns. Specifically, this study aimed to determine the prevalence of reporting sexual concerns, pain with sex, loss of libido, and concerns with unprotected sex. Assessing sexual health concerns during a health care visit as part of a larger distress screening may be one step toward informing more timely, targeted, age-appropriate sexual health assessment for AYAs.

## Method

### Participants and Procedures

Participants (*N* = 127) were AYA patients (ages 19–39) in active treatment or post-treatment survivorship, who presented to three different outpatient cancer clinics in Fort Worth, Texas for routine medical appointments. At the time of their medical appointment, participants were asked to complete a brief online assessment (five to ten minutes in duration) as part of an ongoing AYA oncology patient needs assessment study. After a brief introduction to the study, patients either declined participation or accessed the informed consent page of the survey. The informed consent detailed research aims, procedures, risks, benefits, confidentiality, and contact information, after which participants declined or provided consent to participate and completed the rest of the survey. Participants were not compensated for their participation. Participants completed surveys via an in-clinic electronic tablet or scanned a Quick Response code on their phone.

This study was approved by the University of Texas Southwestern Medical Center Institutional Review Board. Study data were collected and managed using REDCap electronic data capture tools hosted by UT Southwestern Medical Center and supported by CTSA Grant Number UL1 TR003163 from the National Center for Advancing Translational Science (NCATS), a component of the National Institutes of Health (NIH) [29]. The content is solely the responsibility of the authors and does not necessarily represent the official views of the NIH.

### Measures

Consenting patients completed (1) an AYA-specific adapted version of the National Comprehensive Cancer Network (NCCN) Distress Thermometer [30] and (2) Problem List called the Adolescent and Young Adult Psycho-Oncology Screening Tool (AYA-POST) [31], (3) Adolescent and Young Adult Survivorship Psycho-Oncology Screening Tool (AYA-S-POST) [32], and (4) a demographic questionnaire. The AYA-adapted problem list includes the following domains of concern: Practical, Social, Emotional, and Physical. Sex-related concern items are located within the Physical domain.

Patients in active treatment received the AYA-POST, and those in post-treatment survivorship completed the AYA-SPOST. The AYA-POST [33] is a validated screening tool to assess the psychosocial well-being of AYAs with cancer in active treatment and includes a Distress Thermometer (DT) as well as a Needs Assessment Checklist (AYA-NA) that was adapted by Canteen from the NCCN. The AYA-SPOST is a derivative of the AYA-POST and is currently undergoing data collection for validation [33]. The AYA-SPOST also consists of a DT and AYA-NA with items that are specific to AYAs in survivorship. The DT was used to screen patients’ level of distress over the last week for patients in active treatment and survivorship, ranging from zero (no distress) to ten (high distress). All participants were emailed mental health referral information upon completion of the survey.

Patients completed the AYA-NA, which includes potential areas of concern that patients can select. Patients in active treatment endorsed items of distress over the past week and patients in survivorship endorsed items of distress over the past month. In the current study, prevalence of sexual concerns was identified by participants endorsing at least one of the following items on the AYA-POST checklist: Sexual Concerns, Loss of Libido, and Pain with Sex or at least one of the following items on the AYA-SPOST checklist: Sexual Concerns or Unprotected Sex. While the NCCN Distress Thermometer has one generic sexual health item, these items are specific to the AYA-POST and AYA-SPOST.

The demographic survey included questions regarding basic demographic information (e.g., age, biological sex, race, ethnicity, gender identity, income) and cancer history (e.g., cancer diagnosis, stage, date of diagnosis).

### Data Analyses

All data analyses were conducted using IBM SPSS Statistics, version 29 [34]. Sample sizes was determined prior to data collection by referencing a prior study on unmet needs of AYA cancer survivors [35], qualitative interview methods [36], and completion rates (STU-2020–1250). Voided data due to later-found ineligibility and revoked consent was removed, and three survivors’ data were removed due to mistakenly completing the AYA-POST (i.e., active treatment survey).

## Results

Table 1 summarizes the age, gender identity, race, ethnicity, as well as cancer diagnosis and staging information of the overall sample, active treatment, and survivorship patients. Due to the differences in questionnaires completed by participants in active treatment (AYA-POST) and survivorship (AYA-SPOST), results are summarized below by treatment status (Table 2).

A little over one quarter (27.6%, *n* = 35) of all participants (*N* = 127, *M*_*age*_ = 31.96, *SD* = 5.33) endorsed having at least one sexual health concern (i.e., sexual concern, loss of libido, pain with sex, and unprotected sex). Specifically, 31.9% (*n* = 23) of those in active treatment and 21.8% (*n* = 12) of those in survivorship had at least one sexual concern. It should be noted that participants in active treatment had three questions on sexual concerns and survivors only had two, which may contribute to the prevalence rates differences of these concerns across treatment status.

**Figure 1:**
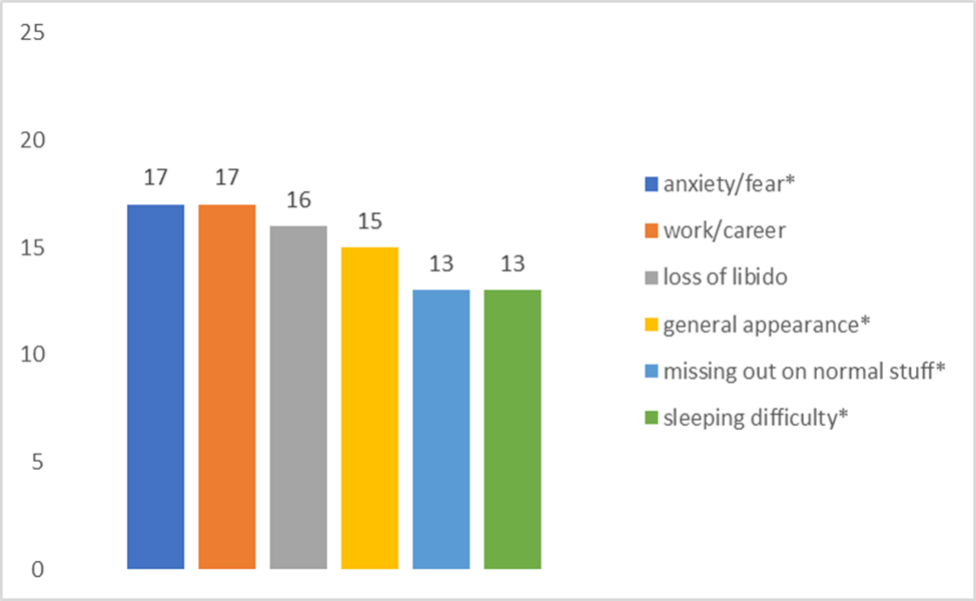
Top Concerns among Active Treatment Participants (n = 23) with Sexual Concerns *Note*. * denotes shared top concerns with active treatment participants without sexual concerns.

**Figure 2:**
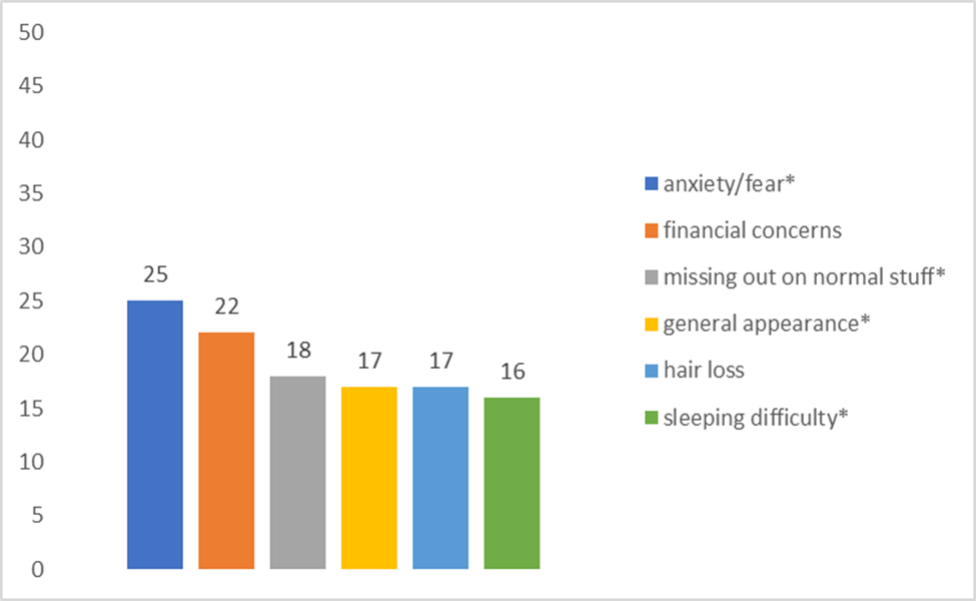
Top Concerns among Active Treatment Participants (n = 49) without Sexual Concerns *Note*. * denotes shared top concerns with active treatment participants with sexual concerns.

**Figure 3:**
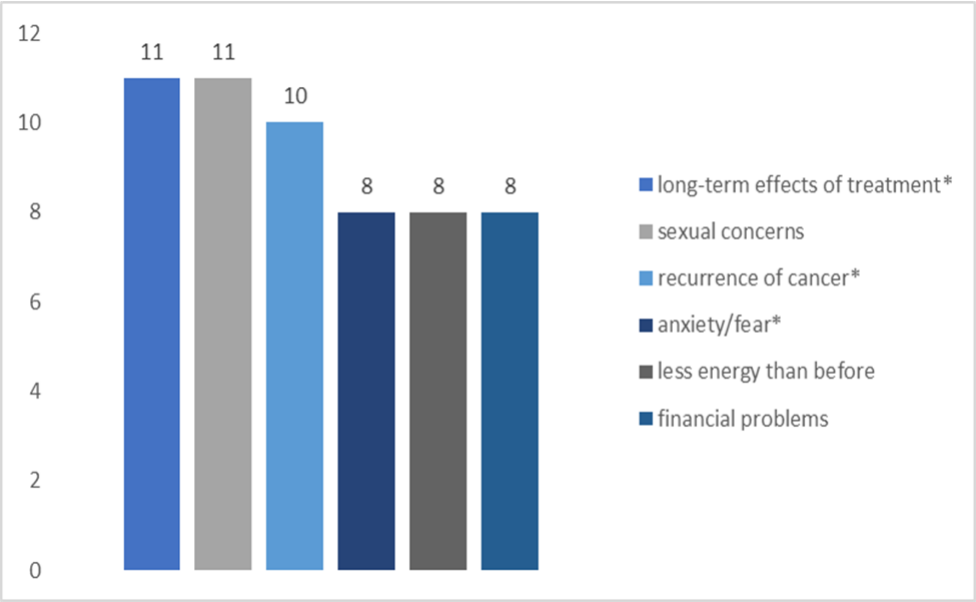
Top Concerns among Survivorship Participants (n = 12) with Sexual Concerns *Note*. * denotes shared top concerns with survivorship participants without sexual concerns.

**Figure 4:**
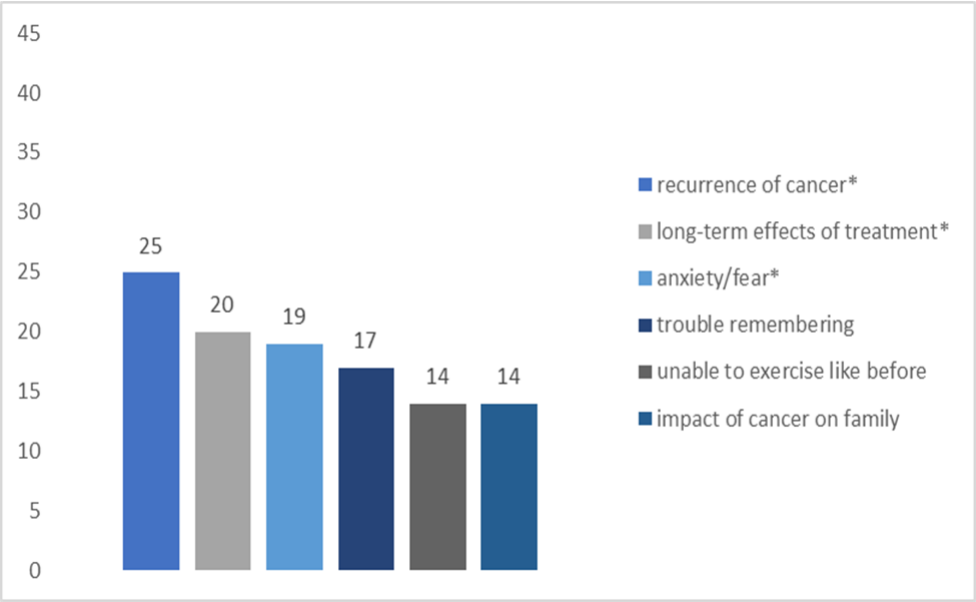
Top Concerns among Survivorship Participants (n = 43) without Sexual Concerns *Note*. * denotes shared top concerns with survivorship participants with sexual concerns.

Figures 1–4 summarize the top concerns among participants with and without at least one sexual concern, separated by treatment status. Independent samples *t*-tests revealed that participants in active treatment who had at least one sexual concern had significantly higher distress (*M* = 5.22, *SD* = 1.93) than those without sexual concerns (*M* = 3.96, *SD* = 2.48; *t*(70) = 2.14, *p* = 0.018). In contrast, distress of participants in survivorship with at least one sexual concern (*M* = 4.75, *SD* = 2.42) did not differ significantly from survivors without sexual concerns (*M* = 3.72, *SD* = 2.91; *t*(53) = 1.12, *p* = 0.134).

More specifically, comparisons of shared top concerns among active treatment participants who reported at least one sexual concern vs no sexual concerns were conducted with chi-square tests of independence. Results revealed significant relations between sexual concerns and general appearance (*χ2* (1, *N* = 72) = 5.91, *p* = 0.015). In contrast, there were non-significant relations between sexual concerns and anxiety/fear (*χ2* (1, *N* = 72) = 3.38, *p* = 0.066), and sexual concerns and sleep concerns (*χ2* (1, *N* = 72) = 3.37, *p* = 0.054).

Among survivors, results showed significant relations between sexual concerns and long-term effects of treatment (*χ2* (1, *N* = 55) = 6.67, *p* = 0.010). Relations between sexual concerns and recurrence of cancer (χ2 (1, *N* = 55) = 1.96, *p* = 0.161), sexual concerns and anxiety/fear (*χ2* (1, *N* = 55) = 1.16, *p* = 0.281) were not significant.

Of the 127 participants (85 female, 40 male, 2 preferred not to answer), 24 (28.2%) females reported at least one sexual health concern, with 15 in active treatment and 9 in survivorship. 10 males (25.0%) endorsed at least one sexual health concern, with 7 in active treatment and 3 in survivorship. Breast cancer (*n* = 14), lymphoma (*n* = 4), stage II were the most common cancer types and stage among females who endorsed at least one sexual concern, whereas the testicular cancer (*n* = 3), lymphoma (*n* = 3), and stage II were the most common cancer types and stage among males who endorsed at least one sexual concern.

## Discussion

The often under-assessed and addressed sexual health concerns among AYA oncology populations emphasize a need to use tools designed for AYAs and an integration of these assessments into routine visits. Results from the current study highlight the prevalence rates of sexual health concerns among a sample of AYAs ages 18 to 39 in both active treatment and survivorship using a needs assessment screener designed specifically for AYAs. The current sample of AYAs in active treatment endorsed sexual concerns at a higher rate (32%) than prevalence findings from a multi-national validation study of the AYA-POST (10%; [33]). These differences warrant further investigation and could be due to a number of factors as the current study included a larger and older age range and was from a single US-based site. The current study found slightly lower prevalence rates of sexual concerns (approximately one third) when compared to some studies of AYAs in the United States. In a study by Acquati and colleagues [12], 52% of AYAs reported sexual dysfunction measured by the Medical Outcomes Survey Sexual Functioning Scale, which was not specifically developed for AYAs [26]. Another found 40–59% of AYAs endorsed sexual dysfunction, with 40% representing ages 15–20, 58% representing ages 20–29, and 59% representing ages 30–39 [37]. Their study used a modified version of the Life Impact Scale, which was originally developed for adult survivors of aggressive non-Hodgkin’s Lymphoma [38]. Another study using the Life Impact Scale [38] showed cancer had a negative impact on 59% AYA’s sexual functioning one year post-diagnosis and 43% at two years post-diagnosis [14]. Conceptualizing the varied prevalence is somewhat difficult given the different measures used among studies.

A recent scoping review of AYA post-treatment survivors identified the most commonly endorsed themes of sexual health concerns were sexual dysfunction, relationship factors, and body image [28]. The current study found that AYAs in active treatment were significantly more likely to endorse general appearance as a concern versus those who did not endorse a sexual health concern; however, this finding was not replicated among AYAs in survivorship. Also contrary to recent findings, AYAs in active treatment or survivorship did not endorse worry about a romantic relationship as a top concern. Differences in assessment methods techniques may be a reason for this discrepancy; specifically, the current study used a brief AYA cancer screener to identify sexual concerns, whereas Cherven and colleagues [28] reviewed a majority of studies that administered a specific measure of sexual functioning.

Comparisons of these outcomes highlight a familiar researcher concern of sensitivity versus specificity. Using a brief screening measure, such as the AYA-POST or AYA-SPOST (similar to the NCCN Distress Thermometer and Checklist), can add to the utility of a medical clinic visit and place fewer demands on a system than a more in-depth diagnostic measure. The screening tool casts a wide net and attempts to capture any possible sources of unmet needs or concerns so that care can be triaged to an appropriate provider as needed. However, such screening tools lack the appropriate specificity to likely identify predictors and correlates of sexual concerns, which leads to continued lack of timely and appropriate addressing of psychosexual needs.

### Clinical Implications

The current study examined the prevalence rates of AYA sexual health concerns using an age-appropriate adaptation of the NCCN Distress Thermometer and Checklist that can be integrated into routine medical care. Results demonstrated that endorsement of at least one sexual concern using this broad screening tool developed specifically for AYAs were comparable to studies measuring sexual concerns. The majority of AYAs with cancer will receive care in a general medical oncology community clinic from non-pediatric providers [39], where services are typically not tailored to AYAs. One of the significant challenges to addressing sexual health concerns in active treatment is the pressure for providers to focus care on life-saving and sustaining treatments [40], while AYA patients in post-treatment survivorship face problems with service delivery such as access to follow-up care [41]. The challenge with assessment of sexual health concerns for AYAs in active treatment and survivorship becomes “how can this be integrated into routine adult medical oncology care?” And once these sexual concerns are identified, “how can it be addressed in an effective and timely manner?” and “will AYAs use interventions and can access them?” Based on prevalence rates reinforced with current study, it is important for providers and support personnel to receive training and information about available AYA-specific sexual health assessments, sexual health interventions, resources, and referrals, and communicate and educate patients throughout the care continuum [7].

Early assessment and integration of sexual health discussion and education into oncology visits may be particularly helpful. A study found 66% of women under 50 with breast and gynecological cancer preferred having written material before having a discussion with their provider about sexual health [7]. Patients also have expressed desire for physicians to use a tailored approach to provide early education and conversations about sexual health, related concerns like body image, and mitigation strategies [42].

Interventions for cancer-related sexual dysfunction in females may include complementary and alternative medicine, pelvic floor physical therapy, mechanical devices to stimulate blood flow to the clitoris or stretch the vaginal wall, hormone therapy, vaginal moisturizers, psychoeducation and psychotherapy [43]. Interventions for males may include mechanical devices, injections, or oral medication to enhance erection, in addition to psychoeducation and psychotherapy [43].

### Limitations

The current study is limited by its homogenous sample in race, ethnicity, and gender identity and thus, generalizability. This is particularly relevant as those belonging in more heterogeneous cultural groups or have an intersectionality of identities may experience more barriers to voice concerns about sexual health or being assessed for sexual concerns, including racism, mistrust, providers lacking knowledge about unique healthcare needs, providers providing information in non-patient-friendly ways, and being subjected to hostility and discrimination [2], [44].

Another limitation of this study is that two different screening measures were utilized (AYA-POST & AYA-SPOST) depending on treatment status and the sexual health concern items were different on each screening measure. While this allowed for participants to complete a screening measure that was most related to participant unmet needs, it limited our ability to combine and compare the reported sexual concerns of two groups. Lastly, validation studies have confirmed the AYA-POST is a useful and appropriate tool for this population, and the AYA-SPOST is currently undergoing a validation study, but results are not yet available.

### Future Research

Future research could replicate the use of the AYA-POST and AYA-SPOST along with other general oncology screening tools that include a sexual concern item to determine if these tools demonstrate sensitivity for identifying sexual health concerns in multiple settings and across more heterogeneous AYA populations. In addition to screening tools that assess for endorsement of issues, quantitative questions could allow for more sophisticated information on the AYAs’ needs. For example, there are differences within the AYA age range, such as younger AYAs reporting needing help with age-incongruent sexual changes (e.g., early menopause) and interruption to development of sexual identity [11], and older AYA survivors reporting more sexual dysfunction [28]. Providing AYAs with opportunities to expand on their specific issues with sexual health and functioning may capture important nuances that inform care.

Additional research could also utilize implementation science to examine how providers can most effectively integrate intervention of sexual health concerns into a medical oncology appointment and identify an appropriate referral based on a patient’s need. Furthermore, this research could examine providers’ initial beliefs about assessing and addressing sexual concerns and compare this with provider perceptions of implementing sexual health intervention and how it impacts effective oncology care.

Further, future studies could continue to utilize sexual health specific screeners that identify common concerns for patients in active treatment vs. post-treatment survivorship. This screening data could be utilized to help develop and fine tune the implementation of evidence-based interventions based on treatment status. For example, patients in active treatment and survivorship are known to experience sexual dysfunction. Certain cancer treatments, such as chemotherapy, are associated with immediate side effects that impact sexual functioning (e.g., lower libido) but may resolve after treatment is complete [45]. Intervention for patients in active treatment may focus on psychoeducation and utilizing alternative strategies to build intimacy with their partner while patients in post-treatment survivorship may desire more active medical appointments like pelvic floor physical therapy to address a similar concern.

## Conclusion

The current study supports previous research that calls for “routine systematic screening to assess sexual function in AYA cancer survivors” [10] and highlights that AYAs in active treatment experience sexual health concerns as well. AYA-specific screening tools that include sexual health concerns can be integrated into the medical visits as recognition that sexual functioning is a part of AYAs’ well-being and quality of life. Addressing these sexual concerns and improving functioning will likely require a multimodal, multi-method approach to better care for AYA oncology populations.

## Figures and Tables

**Table 1:** Summary of Demographic Data and Clinical Characteristics

	Total Sample (*N*=127)		Active Treatment (*n*=72)		Survivorship (*n*=55)	
	*M (SD)*	Range	*M (SD)*	Range	*M (SD)*	Range
**Age**	31.96 (5.33)	19–39	32.33 (5.36)	19–39	31.47 (5.32)	20–39
**Distress Screener**	4.18 (2.58)	0–10	4.36 (2.38)	0–10	3.95 (2.82)	0–10
**Gender Identity**	*n*	%	*n*	%	*n*	%
Female	85	66.9	51	70.8	34	61.8
Male	40	31.5	19	36.4	21	38.2
Prefer not to Answer	2	1.6	2	2.8	0	0
**Race**	*n*	%	*n*	%	*n*	%
White	99	78.0	58	80.6	41	74.5
Black	12	9.4	7	9.7	5	9.1
More than One Race	6	4.7	4	5.6	2	3.6
Asian	5	3.9	1	1.4	4	7.3
Prefer not to Answer	3	2.4	1	1.4	2	3.6
American Indian/Alaska Native	1	0.8	1	1.4	0	0
Native Hawaiian/Other Pacific Islander	1	0.8	0	0	1	1.8
**Ethnicity**	*n*	%	*n*	%	*n*	%
Non Hispanic/Latino	93	73.2	52	72.2	41	74.5
Hispanic/Latino	29	22.8	17	23.6	12	21.8
Prefer not to Answer	3	2.4	2	2.8	1	1.8
Unknown/not reported	2	1.6	1	1.4	1	1.8
**Cancer Diagnosis**	*n*	%	*n*	%	*n*	%
Breast	39	30.7	23	31.9	16	29.1
Other	24	18.9	18	25.0	6	10.9
Lymphoma	15	11.8	7	9.7	8	14.5
Testicular	12	9.4	3	4.2	9	16.4
Leukemia	11	8.7	4	5.6	7	12.7
Brain/CNS Tumor	6	4.7	5	6.9	1	1.8
Sarcoma	5	3.9	3	4.2	2	3.6
Cervical	4	3.1	2	2.8	2	3.6
Melanoma	4	3.1	2	2.8	2	3.6
Colorectal	4	3.1	3	4.2	1	1.8
Prefer not to Answer	2	1.6	2	2.8	0	0
Thyroid	1	0.8	0	0	1	1.8
**Staging**	*n*	%	*n*	%	*n*	%
Unknown/Unstaged	38	29.9	19	26.4	19	34.5
IV	29	22.8	22	30.6	7	12.7
III	25	19.7	11	15.3	14	25.5
II	19	15.0	11	15.3	8	14.5
I	15	11.8	9	12.5	6	10.9
Prefer not to Answer	1	0.8	0	0	1	1.8

**Table 2:** Summary of Sex Concerns by Treatment Status

Concerns	Active Treatment (*n*=23)		Survivorship (*n*=12)	
	Yes	%	Yes	%
Loss of libido	16	69.6	-	-
Sexual concerns	12	52.2	11	91.7
Pain with sex	5	21.7	-	-
Sex/unprotected sex	-	-	3	25.0

*Note.* Proportion is based on number of active treatment vs. survivorship participants who endorsed at least one sexual concern. “−” indicates data that was not collected due to differences between questionnaires for active treatment (AYA-POST) and survivorship (AYA-SPOST).
